# Effects of Temporary Interruption of Hepatic Blood Circulation on the Liver, Brain, and Heart During Experimental Cholestasis

**DOI:** 10.7759/cureus.105014

**Published:** 2026-03-10

**Authors:** Ketevan Kimadze, Tamar Turmanidze, Ketevan Jandieri, Leila Jandieri, Manana Ramishivili, Levan Benashvili, Revaz Otarashvili, Evgeni Asatiani, Liana Kikalishvili

**Affiliations:** 1 Department of Clinical Anatomy and Operative Surgery, Tbilisi State Medical University, Tbilisi, GEO; 2 Department of Anatomy, tbilisi state medical university, tbilisi, GEO

**Keywords:** bile duct, cholestasis, heart, ischemia-reperfusion syndrome, liver

## Abstract

Large-scale liver surgeries often require the temporary interruption of hepatic blood circulation, a process that can lead to potentially irreversible damage to the liver and other organs. This study aims to investigate the histochemical, histological, and ultrastructural changes in the liver and other organs following temporary hepatic blood flow interruption.

Experiments were conducted on 42 rats, randomly assigned to two groups: Group 1 (the animals of this group were subjected to only ligation of the hepatoduodenal ligament; 21 rats) and Group 2 (which underwent bile duct ligation; 21 rats). On Day 6, the hepatoduodenal ligament was interrupted. Decapitation was performed at specific time points following ligature removal: 15 minutes (n=7), 24 hours (n=7), and 48 hours (n=7). Tissue samples were collected for histochemical, histological, and electron microscopic analysis.

The investigation revealed that damage to the liver and other organs after temporary hepatic blood flow interruption and subsequent restoration was more prolonged and severe in the presence of cholestasis than in its absence. In the case of underlying cholestasis after temporary disconnection of the liver from the blood circulation and its restoration at different times, the damaging changes in the liver, heart, and brain lasted longer and were more severe than without cholestasis.

Changes in the liver were already visible in the immediate period after the restoration of blood circulation (15 minutes): new formation of bile ducts, or neoductulogenesis, was observed. At 24 and 48 hours, the number of "neoductus" decreased, possibly due to improved passage and drainage of congested bile through newly formed pathways and reduced pressure in the intrahepatic bile ducts.

The organ-specific nature of injury is evident in that, under conditions of cholestasis, tissue damage develops earlier following the restoration of hepatic blood flow. In the early period after tourniquet removal, pronounced hepatic alterations are observed; although these changes partially regress over time, complete recovery is not achieved. Subsequently, the site of pathological processes shifts from the liver to other vital organs, including the central nervous system and the heart.

## Introduction

The progressive development of liver surgery is historically associated with the improvement of methods for stopping bleeding from the liver during surgical intervention. Performing an operation on the liver requires temporary disconnection of this organ from blood circulation, leading to damaging changes in both the liver and other organs that can be irreversible, in some cases [1-5].

Therefore, changes in the liver and other organs have been studied, in particular the intestines, pancreas, and spleen, where significant damaging changes develop at different times after temporary disconnection of the hepatic blood circulation and restoration of blood circulation [6].

The presence of cholestasis significantly aggravates damaging changes in both the liver and extrahepatic organs, which reflect the nature of the dynamics of damaging and reparative processes. The process of organ damage leads to observable changes, that is, in the shortest time after the restoration of blood circulation to the liver, damaging changes are observed in all organs, which gradually undergo elimination after the tourniquet is removed. But changes in various organs begin at distinct times, damage develops with unequal severity, and both the damage and the rehabilitation process require different durations [2,3,6].

The brain and heart belong to a special category of organs, since the structural and functional changes in these organs determine the degree and severity of the violation of the body's homeokinesis and its reversibility [7,8].

The main goal of our research is to study histological, histochemical, and ultrastructural changes that develop in the liver, brain, and heart after temporary disconnection of the liver from the blood circulation and restoration of blood circulation at the 15th, 24th, and 48th hours under conditions of cholestasis.

## Materials and methods

Study design

The experiment was carried out at the research base of Tbilisi State Medical University. The study was conducted on white Wistar rats (a total of 42). All rats were housed under standard laboratory conditions with access to food and water. The study protocol was approved by the institutional ethical committee. All procedures complied with ARRIVE guidelines [9] for animals used for scientific purposes. The sample size (n=42) was determined based on power calculations and previous experimental studies evaluating hepatic ischemia-reperfusion surgery. This number was sufficient to detect significant differences in morphological and ultrastructural changes across the selected time points [10].

Group 1

The animals of this group were subjected to ligation of the hepatoduodenal ligament (10 minutes). After its removal, at the 15th minute (7 animals), at the 24th hour (7 animals), and at the 48th hour (7 animals), the animals were decapitated, and materials were collected for the appropriate methods (histological, histochemical, and electron microscopy).

Group 2 

The animals of this group were subjected to ligation of the bile duct (for modeling the cholestasis). On Day 6 after ligation, a knot was applied to the hepatoduodenal ligament. After its removal, at the 15th minute (7 animals), 24th hour (7 animals), and 48th hour (7 animals), we decapitated the animals and collected material for the appropriate methods (histological, histochemical, and electron microscopy).

Surgical procedure

Cholestasis was induced as follows: rats were anesthetized with isoflurane, using 5% for induction and 2-3% for maintenance in oxygen, while normothermia (37 °C) was maintained with a heating pad. The abdominal hair was shaved, and the operative area was cleaned with antiseptic solution (10% povidone-iodine) followed by 70% ethanol. A midline laparotomy (~4 cm) was performed along the linea alba using surgical scissors. The abdominal cavity was gently retracted, and the liver was exposed by gently retracting the intestines caudally.

The common bile duct was identified and isolated. 0.25% Novocain was locally applied to the hepatoduodenal ligament. Using fine micro-forceps, the bile duct was carefully separated from the portal vein and hepatic artery. Two 6-0 silk ligatures were placed around the bile duct, after which the duct was transected between the ligatures. The abdominal cavity was closed in layers with interrupted sutures. Successful induction of cholestasis was verified by visible dilation of the bile duct [11,12].

Tissue collection and processing

To assess the general morphological changes of the organs, we used the hematoxylin and eosin staining method, which allowed us to identify pathological changes and processes at the tissue and cellular level, in particular dystrophic, necrotic, dyscirculatory, etc.

To assess the ultrastructural basis of cellular damage and its rehabilitation processes, we used transmission electron microscopy, which allowed us to assess the state of membrane structures and intercellular connections.

Electron Microscopy (Ultrastructural Examination)

Following routine processing, tissue specimens were embedded in Epon and polymerized for 48 hours at 60 °C. Ultrathin sections were prepared using an LKB or LKB III ultramicrotome (Leica Microsystems, Wetzlar, Germany) and placed on palladium grids. Contrast staining was achieved by staining with 5% uranyl acetate in absolute methanol (for 15 minutes at 45 °C), followed by Reynolds’ lead citrate. Ultrastructural analysis was performed using Tesla-100 (TESCAN, Brno, Czechia), Hitachi-100 (Hitachi High-Tech, Tokyo, Japan), and Tesla-135-500 (TESCAN, Brno, Czechia) electron microscopes.

In order to assess the rapid consequences of hypoxic injuries in liver tissue and the dynamics of the rehabilitation process, we used the Shabadash histochemical reaction to glycogen, since histochemistry provides unique information about the results of cellular metabolism [13].

Histomorphometry in experimental cholestasis and in conditions without cholestasis was used to analyze the proliferation of bile ducts. The study evaluated the area of the bile ducts, their lumens, and the epithelial cells of the bile duct mucosa. The ratio of epithelial cells to the duct was calculated by subtracting the luminal area from the total bile duct area and dividing by the epitheliocyte area [14,15].

\(\text{Ratio of cells to bile duct} = \frac{\text{Bile duct area} - \text{Bile duct lumen area}}{\text{Epitheliocyte area}}\)

Statistical analysis

Statistical analyses were performed using GraphPad Prism (GraphPad Software, San Diego, CA). Student’s t-test was used to determine statistical significance, with p-values < 0.05 considered significant.

## Results

Group 1

The main task of this group of experiments was to determine the nature of the changes that develop in the liver and extrahepatic organs without any background liver damage after temporary (10 minutes) ischemia of the liver and restoration of blood circulation. However, such conditions are rarely encountered in practice.

As a result of studying the experimental animals of this group, it was established that after temporary disconnection of the liver from the blood circulation, significant changes develop in the liver and other organs at different times (15 minutes, 24 hours, 48 hours) after restoration of blood circulation, which reflect the dynamics of the damaging reparative processes under these conditions.

Changes That Developed Within 15 Minutes After the Restoration of Blood Circulation

**Figure 1:**
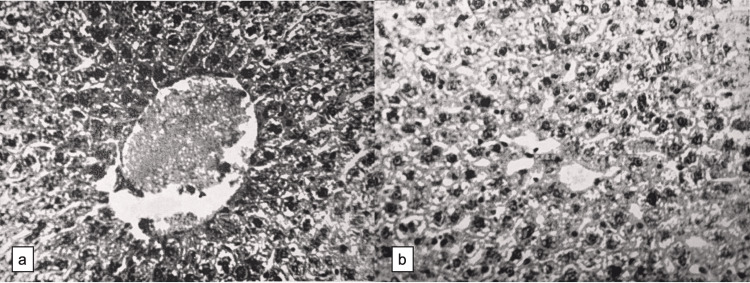
Light microscopy of liver tissue at 15 minutes after restoration of hepatic blood circulation (H&E, ×200) (a) The central vein contains erythrocyte aggregates. (b) Uneven staining of hepatocyte cytoplasm, with alternating basophilic and pale (cleared) areas, giving the cytoplasm a non-homogeneous appearance.

During occlusion of the afferent blood vessels of the liver (ligation of the hepatoduodenal ligament for 10 minutes), congestive phenomena were observed in the portal system. The organs of the gastrointestinal tract were bluish and cyanotic, and the spleen was enlarged five times or more. The liver was gray-chestnut in color. In preparations stained with hematoxylin-eosin, the micromorphological structure of the liver was preserved. Most of the central veins were filled with blood. Congested blood was represented by emptied erythrocytes (Figure 1a-1b). Hepatocytes were swollen. A sharp decrease in the amount of glycogen was noted in hepatocytes.

**Figure 2:**
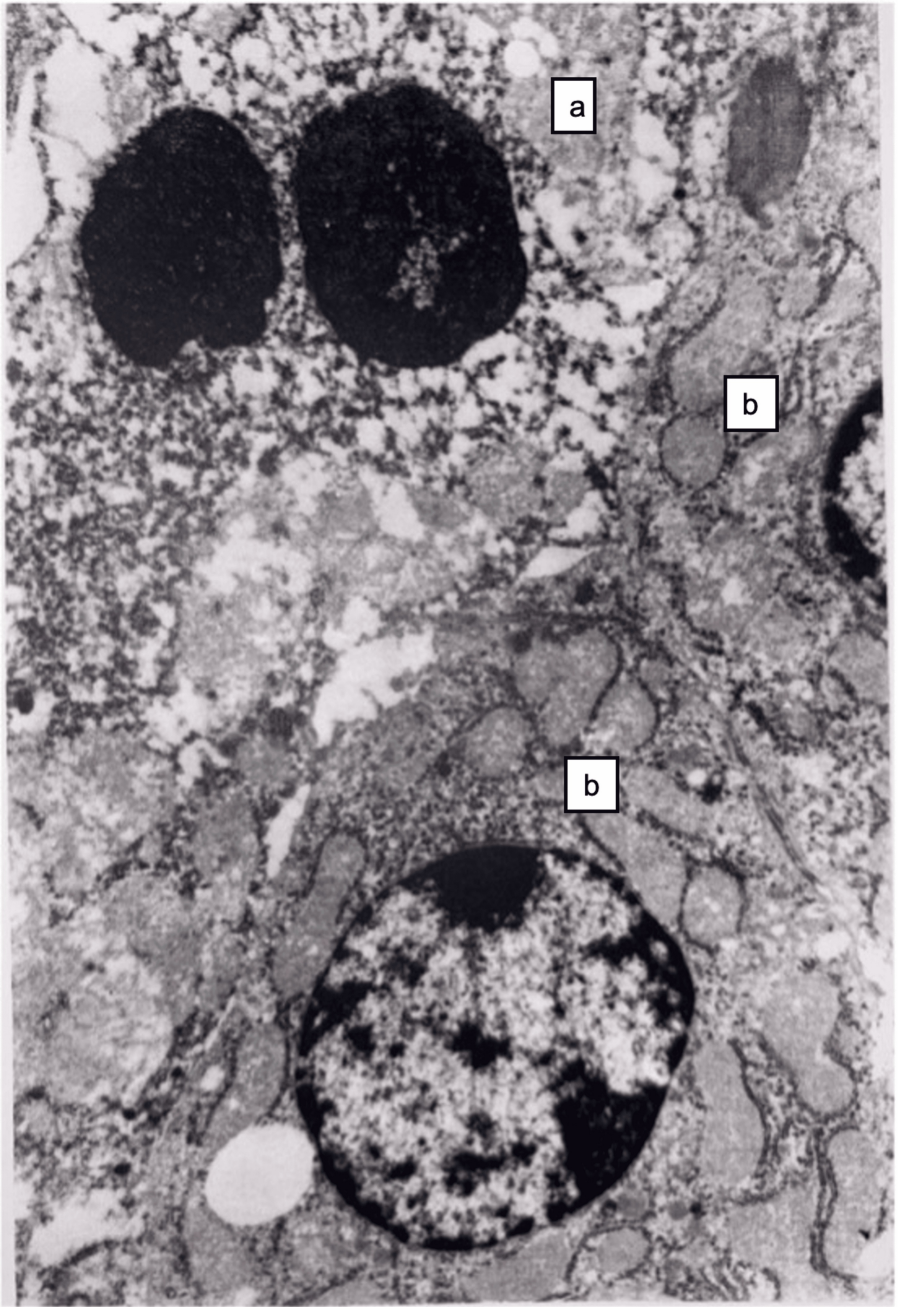
Liver tissue at 15 minutes after restoration of hepatic blood circulation. Necrotic (a) and relatively preserved (b) hepatocytes. In the damaged hepatocytes, nuclei show heterochromatin condensation and fragmentation. The membranes of the endoplasmic reticulum and the plasma membrane are disrupted, and free ribosomes and polysomes are present in the cytoplasm. Desmosomal junctions between adjacent hepatocytes are dissociated. Electron microscopy, ×10,000.

Electron microscopy revealed that the hepatocytes located on the periphery of the lobe were swollen, granular, and non-granular endoplasmic reticulum were evenly developed. Mitochondria were swollen and round or oval in shape. In some areas, dissociation and necrosis of hepatocytes were noted. In the nuclei, chromatin marginalization and heterochromatinization were noted (Figure 2).

**Figure 3:**
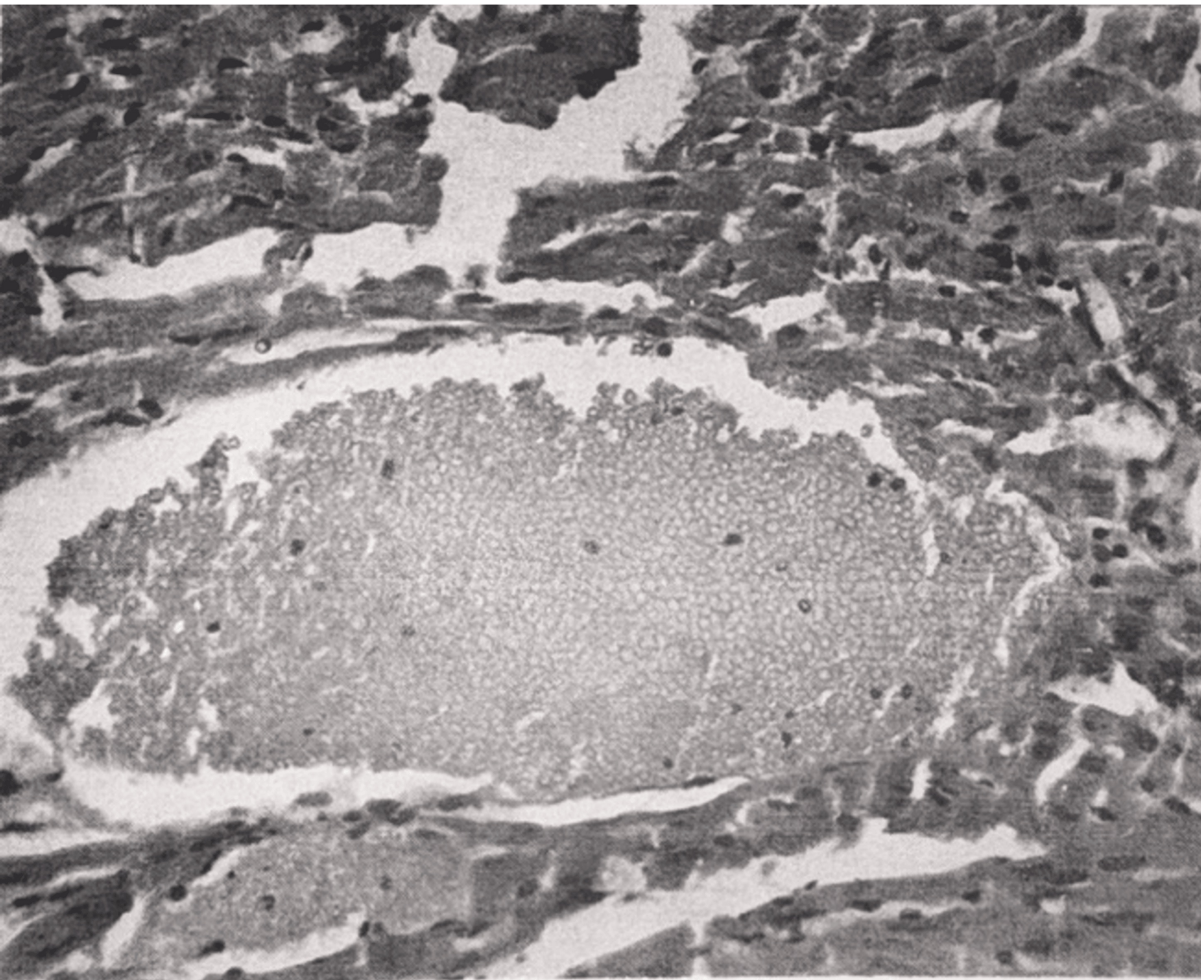
Myocardium at 15 minutes after restoration of hepatic blood circulation. A small blood vessel filled with erythrocytes within the myocardial tissue. Light microscopy, H&E, ×400.

The histological structure of the myocardium was mostly preserved. In some areas, there was hyperemia (Figure 3) and interstitial edema. Foci of fatty dystrophy of myocardiocytes were visible.

**Figure 4:**
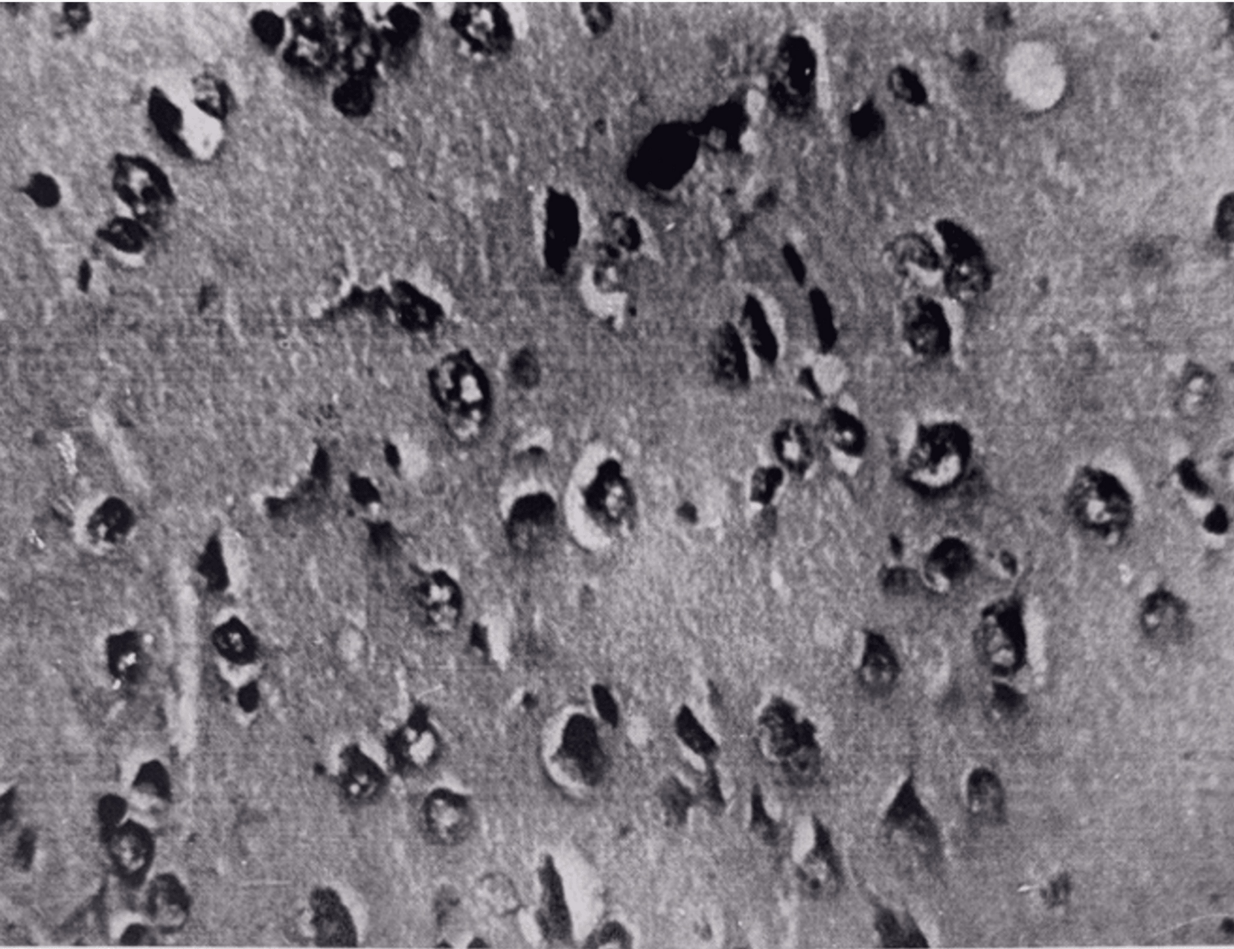
Brain tissue at 15 minutes after restoration of hepatic blood circulation. Pericellular edema in the cerebral cortex. Light microscopy, H&E, ×400.

Electron microscopy revealed swelling of myofibrils in cardiomyocytes. Mitochondria were swollen; ridges were flattened and destroyed in places. In the brain, pronounced pericellular and perivascular edema was observed, and most of the pyramidal neurocytes of the cortex underwent dystrophic, necrobiosis, and necrotic changes. The process of necrophagy was visible (Fig. 4).

Electron microscopy showed numerous mitochondria in the cytoplasm of neurons in various stages of damage, dilation, and degranulation of the cisterns of the rough endoplasmic reticulum. The nuclear envelope of some neurocytes was wrinkled, and its integrity was disrupted.

Changes That Developed Within 24 Hours After the Restoration of Blood Circulation

No noticeable changes were observed in the abdominal cavity of animals dissected 24 hours after occlusion of the afferent blood vessels of the liver. In preparations stained with hematoxylin and eosin, the micromorphological structure of the liver was preserved. Parts of the central veins were still filled with blood. Hepatocytes were swollen. The cytoplasm was slightly basophilic and vacuolated. The spaces of Disse were dilated. A further decrease in glycogen content was observed compared to the 15-minute period.

**Figure 5:**
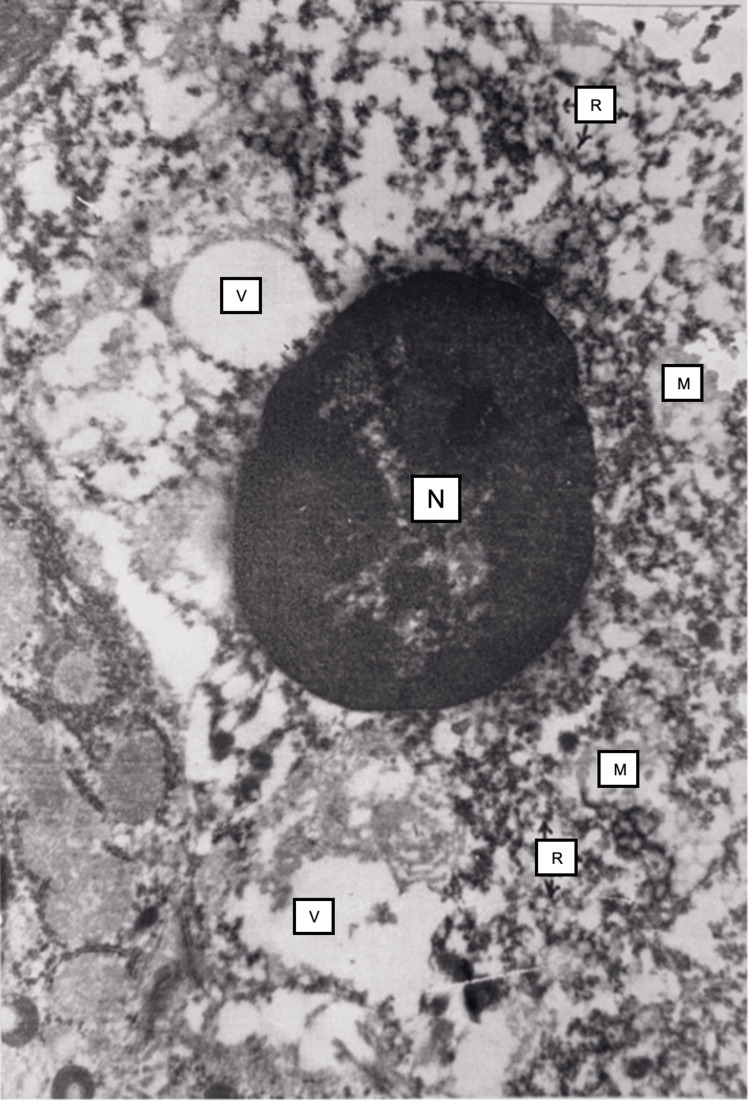
Liver tissue 24 hours after restoration of hepatic blood circulation The nuclei of apoptotic hepatocytes (N) show pyknosis and heterochromatin condensation. The cytoplasm contains vacuoles (v). The endoplasmic reticulum is disrupted, and detached ribosomes (R) are presented in the cytosol. Destructively altered mitochondria (M) are present in the cytoplasm. Electron microscopy, ×15,000

Electron microscopy showed severe ultrastructural changes in hepatocytes located on the periphery of the liver, in particular, severe vacuolization of mitochondria (Fig. 5) and destruction of ridges were observed, the integrity of the membrane of some hepatocytes was disrupted, the nuclear membrane was torn (disrupted), and the contents were mixed with the cytoplasm in the form of structureless electrically dense masses.

The histological structure of the myocardium was mainly preserved. Interstitial edema was observed in some areas, and aggregates of erythrocytes were visible in individual capillaries. In the majority of myocardiocytes, striation was well manifested, glycogen was reduced compared to normal, but slightly increased compared to the 15-minute period.

**Figure 6:**
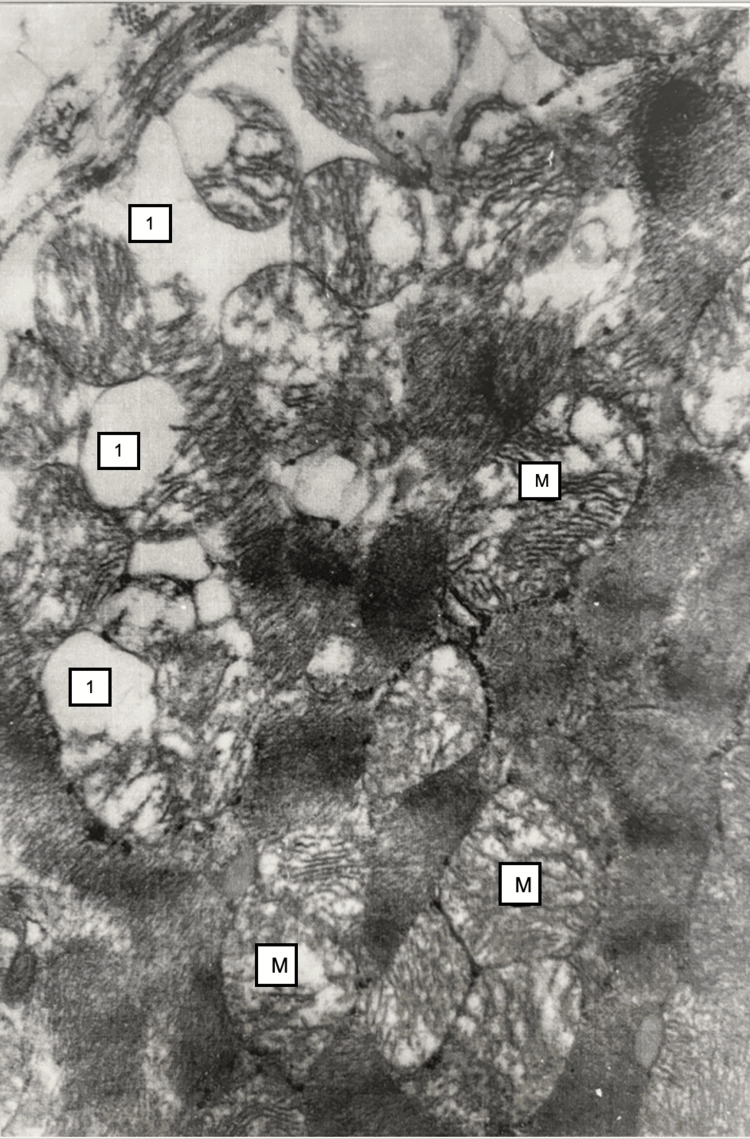
Myocardium, 24 hours after restoration of hepatic blood circulation. Mitochondria (M) cristae structure disruption. The sarcoplasmic reticulum cisternae are dilated (vacuolization) (1). Electron microscopy, ×12,000.

Electron microscopy revealed damaging changes in mitochondria in myocardiocytes, namely, their swelling, shortening, and destruction of ridges. Vacuolization of the sarcoplasmic reticulum (Figure 6).

In the brain, dyscirculatory necrobiosis and necrotic changes were even more pronounced compared to the 15-minute period. Disappearance of the fog substance and chromatolysis were noted. The intensity and spread of the target processes were increased compared to the 15-minute period. Electron microscopy showed predominantly severe damage to the mitochondria of cortical neurons, with vacuolization as well as swelling and destruction of ridges, and transparency of the matrix. Single sinusoidal vesicles were visible in the presynapses.

Changes That Developed Within 48 Hours After the Restoration of Blood Circulation

**Figure 7:**
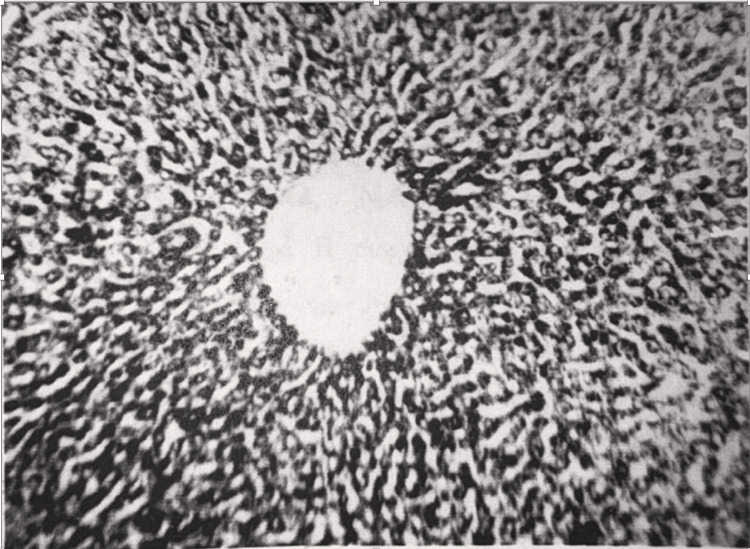
Liver tissue 48 hours after restoration of hepatic blood circulation. The glycogen content in hepatocytes is almost restored to the control level. Light microscopy, ×150.

At autopsy of animals 48 hours after occlusion of hepatic afferent vessels, no noticeable changes were observed in the abdominal cavity. In preparations stained with hematoxylin and eosin, the liver lesions were almost completely rehabilitated. In particular, hepatocytes acquired a normal structure, and the glycogen content in hepatocytes was normalized (Figure 7). Mitosis occurred much more often than normal.

**Figure 8:**
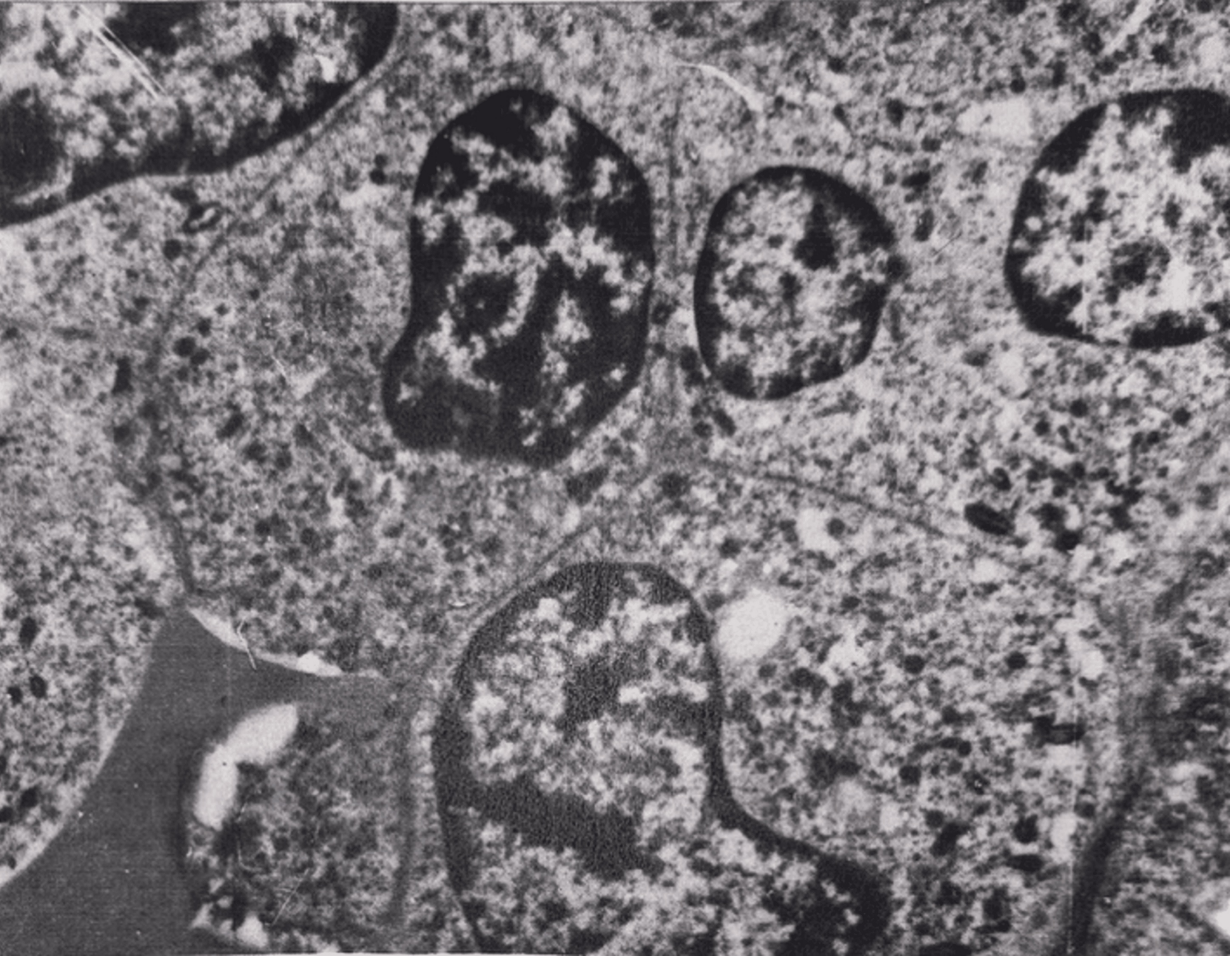
Liver tissue 48 hours after restoration of hepatic blood circulation. Electron-dense glycogen granules in the cytoplasm of hepatocytes. Electron microscopy, ×10,000.

Electron microscopy revealed that the ultrastructural structure of hepatocytes approached the norm. There were clearly distinguished biliary and vascular areas with microactivities. The cytoplasm contained a well-developed granular endoplasmic reticulum, among which electron-dense glycogen granules were visible (Figure 8).

**Figure 9:**
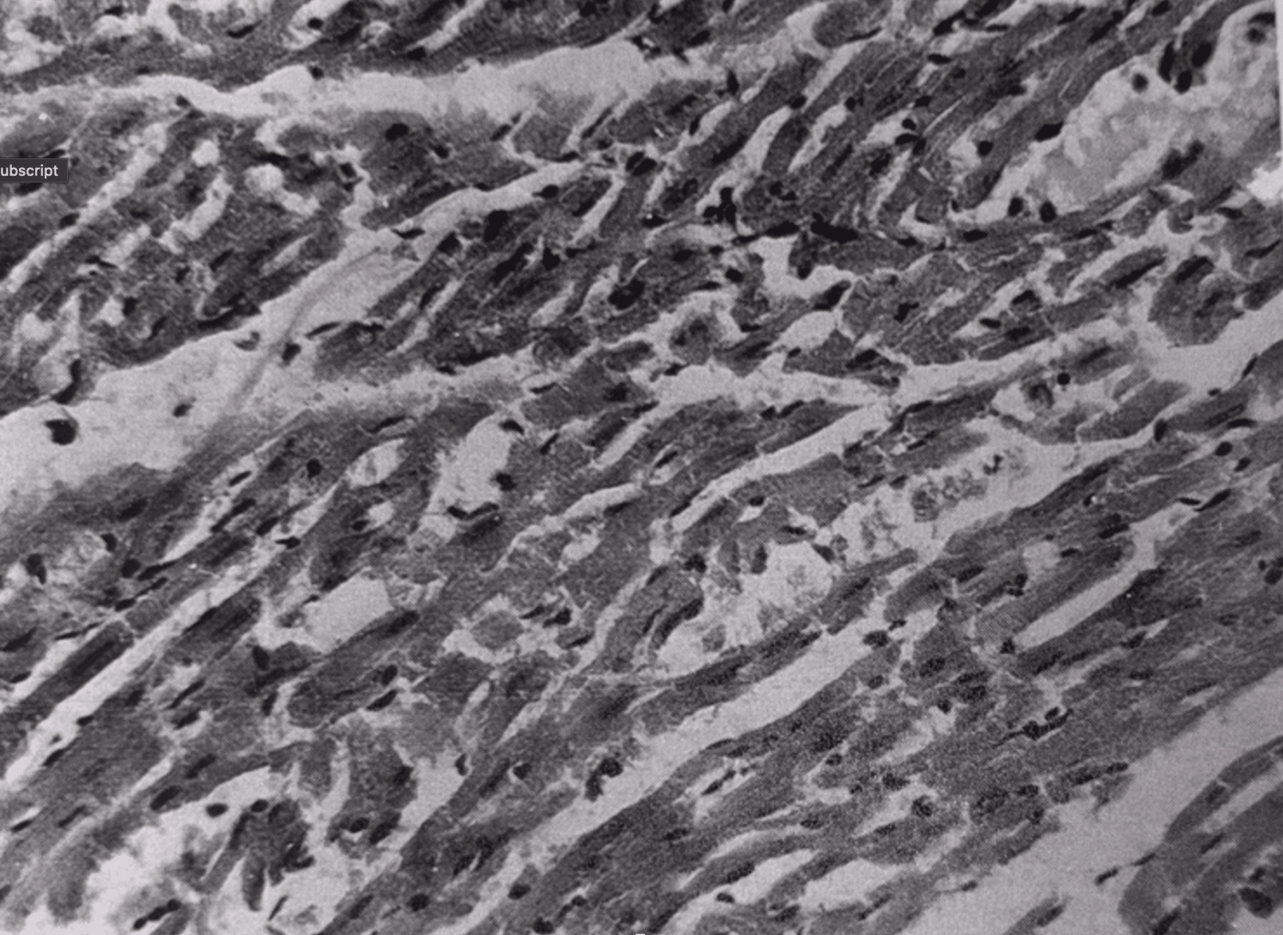
Myocardium, 48 hours after restoration of hepatic blood circulation. Interstitial edema and fragmentation of cardiomyocytes. Light microscopy, H&E, ×240

The histological structure of the myocardium was mainly preserved. In some areas, foci of myocardial swelling and fragmentation were noted (Figure 9); the amount of glycogen was slightly reduced compared to the norm.

**Figure 10:**
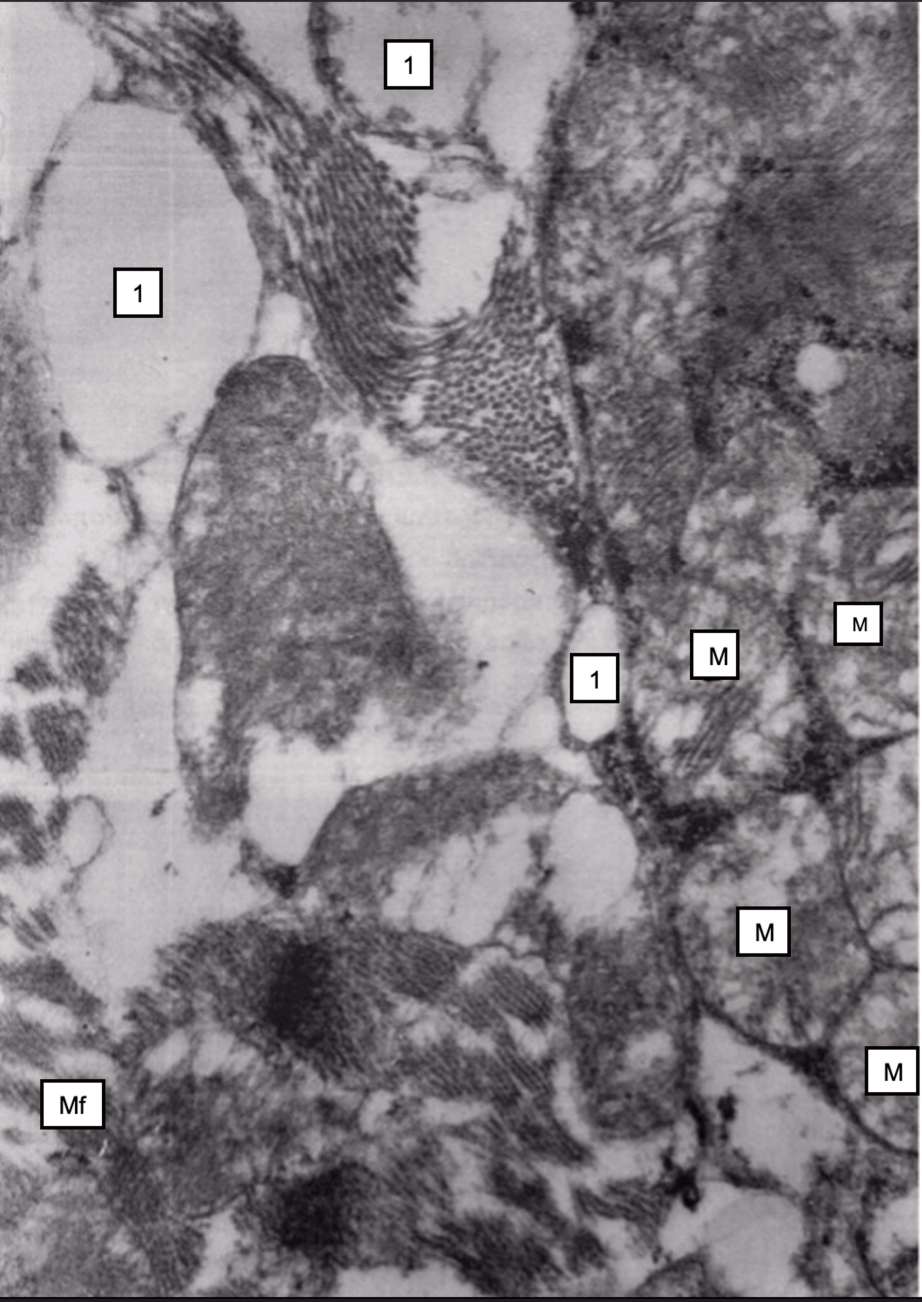
Myocardium, 48 hours after restoration of hepatic blood circulation Mitochondria (M) cristae destruction. The sarcoplasmic reticulum vacuolization (1), and myofibrils (Mf) focal disruption. Electron microscopy, ×24,000.

By electron microscopy, mitochondria in myocardiocytes were swollen, ridges were destroyed, the transparency of the matrix was ​​​​increased, and lipid droplets were visible in some cardiocytes (Figure 10).

**Figure 11:**
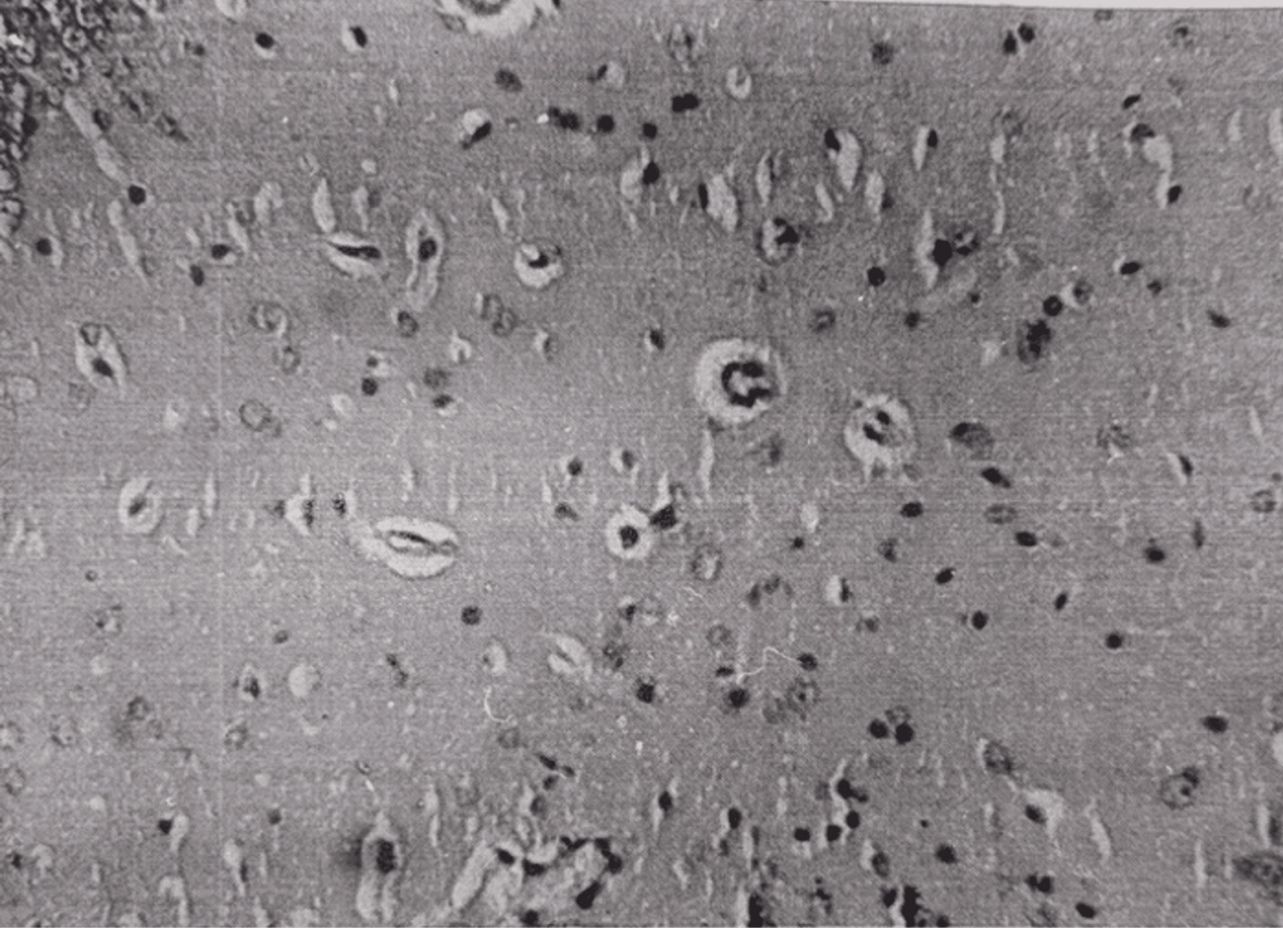
Brain tissue, 48 hours after restoration of hepatic blood circulation. Perivascular and pericellular edema, dystrophy and necrosis of neurons, and neuronal dropout. Light microscopy, H&E, ×240.

In the brain, pronounced pericellular and perivascular edema was observed. Fields of neuronal loss were visible in neurocytes. The process of neurophagy was visible (Figure 11).

Electron microscopy showed that some of the cortical neurocytes underwent lysis, and fragments of cell membranes and organelles were visible in their place. In some neurons, there was disorganization of mitochondria, degranulation of the rough endoplasmic reticulum, and a decrease in the number of synaptic vesicles in presynapses.

Group 2 

Changes That Developed Within 15 Minutes After the Restoration of Blood Circulation

When opening the abdominal cavity, the common bile duct was macroscopically enlarged and filled with bile. The liver was enlarged. After emptying the common bile duct of bile, a tourniquet was applied to the hepato-duodenal ligament for 10 minutes. Congestive phenomena were observed in the portal system. Around 15 minutes after removing the tourniquet, the liver was seen to be purple. The spleen returned to its original size.

In preparations stained with hematoxylin and eosin, the micromorphological structure was preserved, the spaces of Disse were dilated, and the liver hepatocytes underwent large and small droplet fatty dystrophy. In the center and periphery of the lobules, small and relatively large foci of coagulative necrosis were visible, containing eosinophilic detritus infiltrated by leukocytes. Proliferation of bile capillaries, or the so-called "neoductules," was also visible. Glycogen has almost disappeared and was detected only in single hepatocytes, mainly on the periphery of the lobules.

Electron microscopy showed hepatocytes swollen, with dissociation of hepatocytes in some areas. Fat droplets were visible in hepatocytes. Individual pyknotic nuclei and apoptotic necrotic hepatocytes were visible. The spaces of the Disse were expanded, with single erythrocytes visible in them. Single erythrocytes were observed in the central veins. Erythrocyte sludges (clusters) were observed in the central veins.

**Figure 12:**
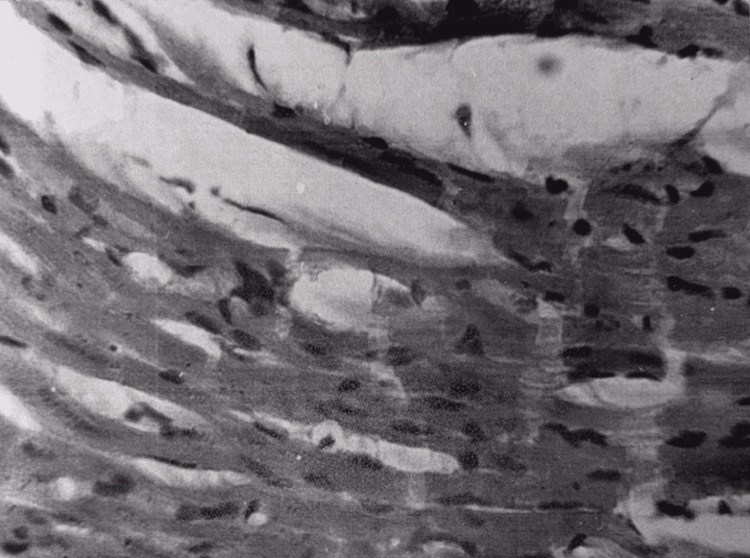
Heart tissue, 15 minutes after restoration of hepatic blood circulation, with underlying cholestasis. Interstitial edema and fragmentation of cardiomyocytes. Light microscopy, H&E, ×480.

The histological structure of the myocardium was mainly preserved. In some areas, there was hyperemia, interstitial edema, and fatty dystrophy of myocardiocytes. In the majority of myocardiocytes, striation was weakly manifested. Fragmentation of myocardiocytes was observed here and there (Figure 12) with small droplets of sudanophilic lipids. A sharp decrease in glycogen was noted.

**Figure 13:**
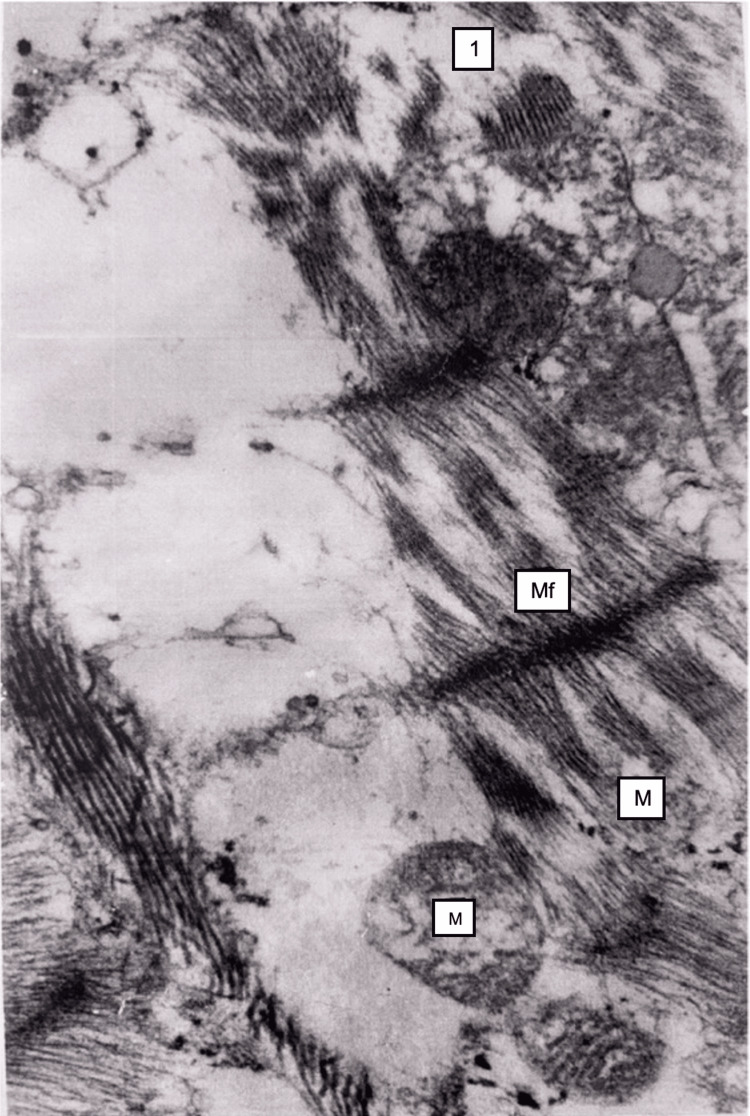
Myocardium, 15 minutes after restoration of hepatic blood circulation, with underlying cholestasis. Destruction of myofibrils (Mf) and mitochondria (M), along with sarcoplasmic vacuolization (1). Electron microscopy, ×14,000.

Electron microscopy revealed swelling of myofibrils and mitochondria in cardiomyocytes, mitochondrial ridges were flattened, and sometimes underwent destruction, and matrix transparency was increased. Sarcoplasmic reticulum cisterns were dilated (Figure 13). Osmophilic lipid droplets were observed in some cardiomyocytes.

**Figure 14:**
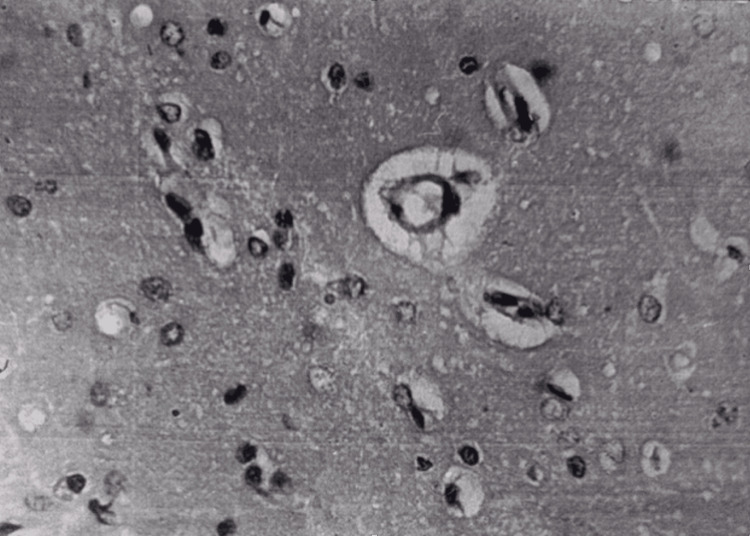
Brain 15 minutes after restoration of hepatic blood circulation, with underlying cholestasis. Perivascular and pericellular edema is observed. Light microscopy, H&E, ×480.

In the brain, pronounced pericellular and perivascular edema was observed (Figure 14). The majority of pyramidal neurocytes of the cortex underwent dystrophic, necrobiosis, and necrotic changes, and the process of neurophagy was visible.

**Figure 15:**
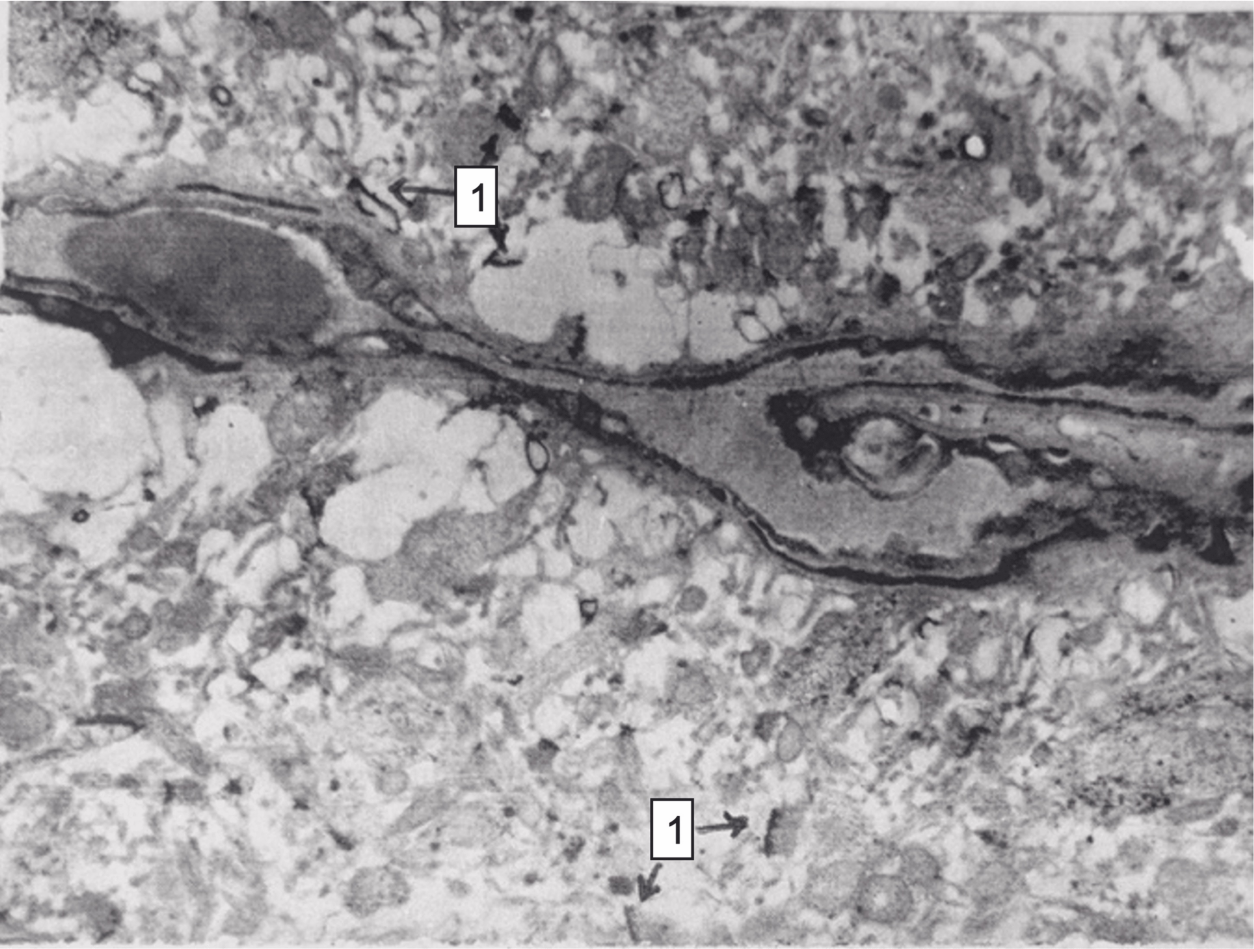
White matter of the brain, 15 minutes after restoration of hepatic blood circulation, with underlying cholestasis. A capillary demonstrates signs of stasis, with pericapillary edema and fragments of synaptic desmosomal structures (1). Electron microscopy, ×10,000.

Electron microscopy showed numerous mitochondria in various stages of damage in the cytoplasm of neurons, dilation and degradation of endoplasmic reticulum cisternae. The nuclear membranes of some neurocytes were wrinkled, and their integrity was disrupted; the number of synaptic vesicles in presynapses was reduced. Most synapses were disintegrated, and only desmotic contacts remain. Perivascularly and pericellularly, severe edema was observed, and stasis phenomena occurred in the capillaries (Figure 15).

Changes That Developed Within 24 Hours After the Restoration of Blood Circulation

In preparations stained with hematoxylin and eosin, the micromorphological structure of the liver was preserved. Disse spaces were dilated, and hepatocytes underwent small and large droplet fatty dystrophy. Foci of necrosis were rarely found. An increase in the number of apoptotic hepatocytes and proliferation of newly formed bile ducts - neoductules - were observed. Mitotic figures were observed quite often. Glycogen almost disappeared in hepatocytes, except for a single hepatocyte, in which glycogen was visible as a trace.

Electron microscopy revealed the dilation of Disse spaces in the liver. In the majority of hepatocytes, severe ultrastructural changes were observed, which were manifested by severe vacuolization of mitochondria and destruction of ridges, vacuolization of endoplasmic reticulum cisternae, and degranulation. Disappearance of glycogen granules was noted.

**Figure 16:**
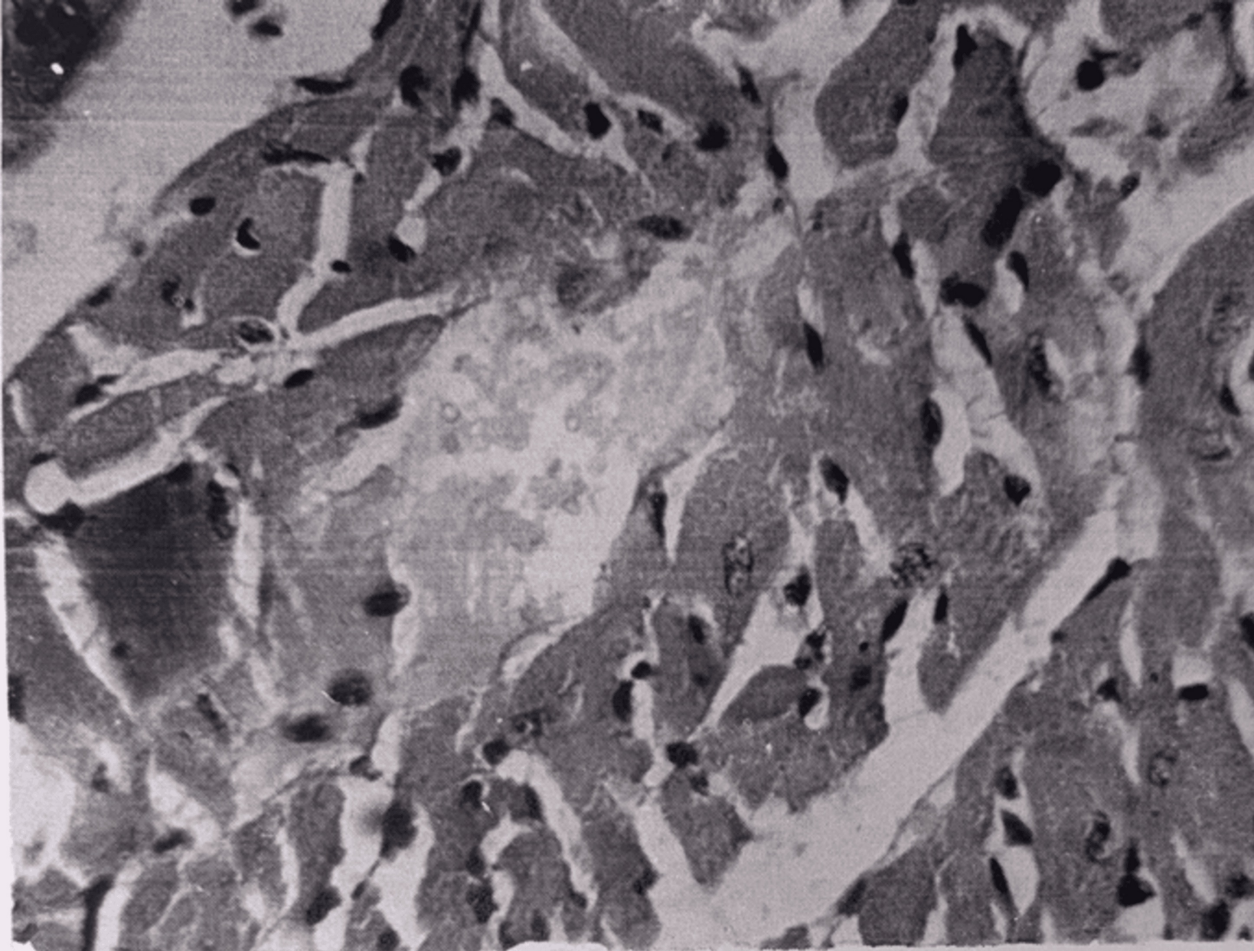
Heart tissue, 24 hours after restoration of hepatic blood circulation, with underlying cholestasis Focal necrosis of cardiomyocytes. Light microscopy, H&E, ×480.

**Figure 17:**
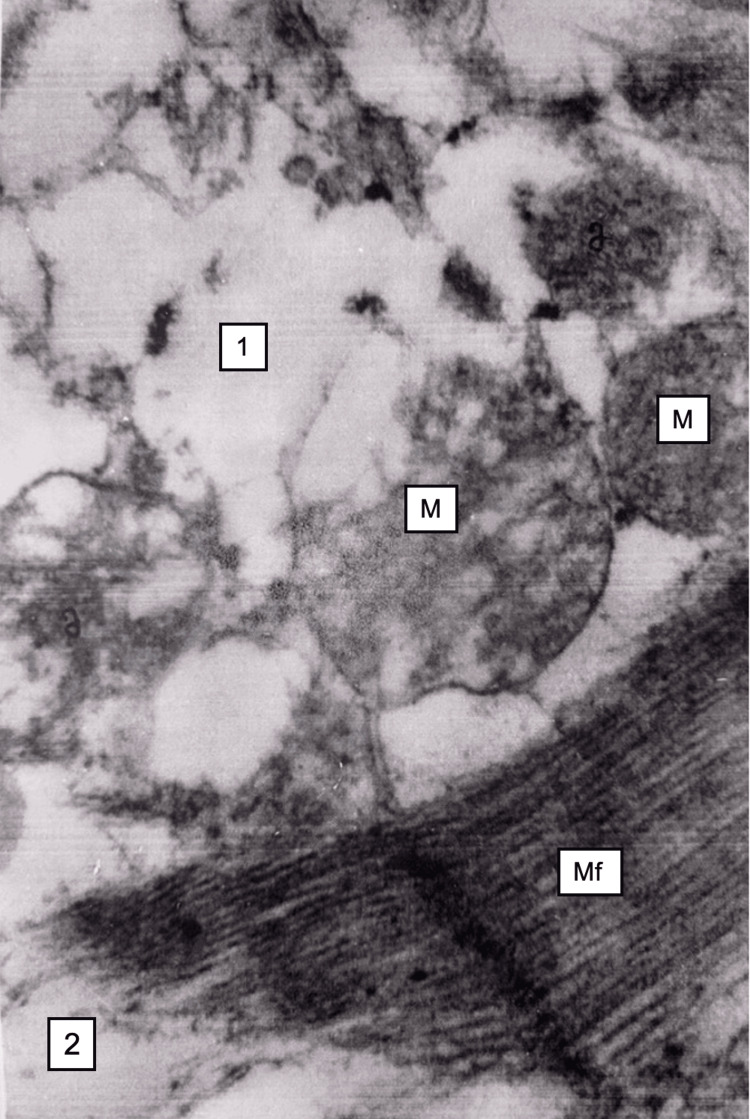
Myocardium, 24 hours after restoration of hepatic blood circulation, with underlying cholestasis Mitochondria show destructive structural changes. Vacuolization of the sarcoplasm (1) and disruption of myofibrils (Mf) (2) are observed. Electron microscopy, ×24,000

The histological structure of the myocardium was mainly preserved. In some areas, interstitial edema was observed, and aggregates of erythrocytes were visible in individual capillaries. Myocardiocytes were swollen. In the majority of myocardiocytes, striation was weakly manifested. Here and there, focal necrosis was visible (Figure 16), with small droplets of sudanophilic lipids. The amount of glycogen was sharply reduced compared to normal, but slightly increased compared to the 15-minute period. Electron microscopy (Figure 17) showed swelling and destruction of mitochondria in myocardiocytes, a decrease in their number and transparency of the matrix, destruction of myofibrils, and vacuolization of the sarcoplasmic reticulum.

**Figure 18:**
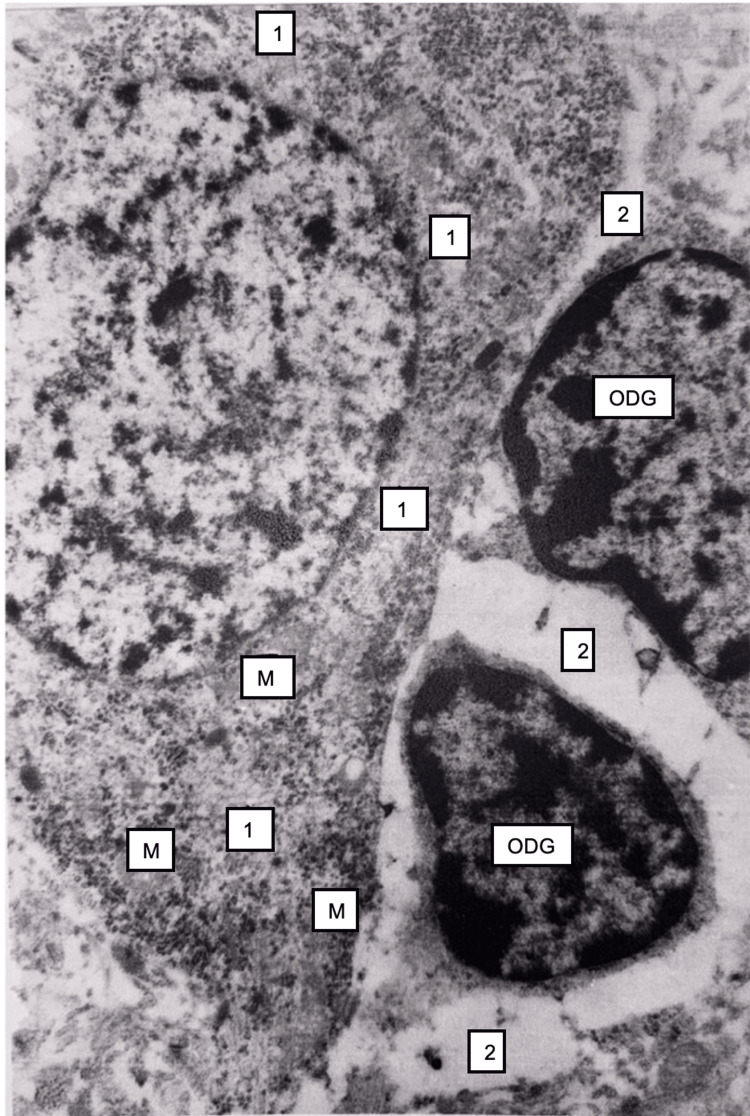
Brain tissue, 24 hours after restoration of hepatic blood circulation, with underlying cholestasis. A pyramidal neuron shows focal areas of chromatolysis in the cytoplasm (1), along with destruction of mitochondria (M). Pericellular edema (2) is present, especially around oligodendrocytes (ODG). Electron microscopy, ×15,000.

In the brain, dyscirculatory, necrobiosis, and necrotic changes were more pronounced in this period compared to the 15-minute period. Neurophagia phenomena were observed, and pericellular edema was pronounced. The majority of pyramidal neurocytes of the cortex underwent dystrophic and necrotic changes. The intensity and spread of damaging processes were increased compared to the 15-minute period. Electron microscopy showed predominantly severe damage to the mitochondria of cortical neurons, vascularization, destruction-fragmentation of their nuclear membranes, and pyknosis. Pericellular edema was expressed around oligodendrogliocytes (Figure 18). The number of vesicles in presynapses was reduced.

Changes That Developed Within 48 Hours After the Restoration of Blood Circulation

In preparations stained with hematoxylin-eosin, liver damage changes were still visible. Some hepatocytes acquired a normal structure. In addition, a decrease in the amount of glycogen was noted in several hepatocytes. Mitoses occurred much more often than in a 24-hour period.

By electron microscopy, biliary and vascular areas were clearly distinguished in hepatocytes with microcavities. The cytoplasm contained a well-developed granular endoplasmic reticulum, the cisterns of which were filled with proteins. Mitochondria were swollen; the cisterns of the endoplasmic reticulum were vacuolated. Chromatin margination was observed in the nuclei; however, the nuclei became indistinct and difficult to identify, dissociation began, and connections were preserved only in the desmosomes in the area of ​​​​the bile capillaries.

**Figure 19:**
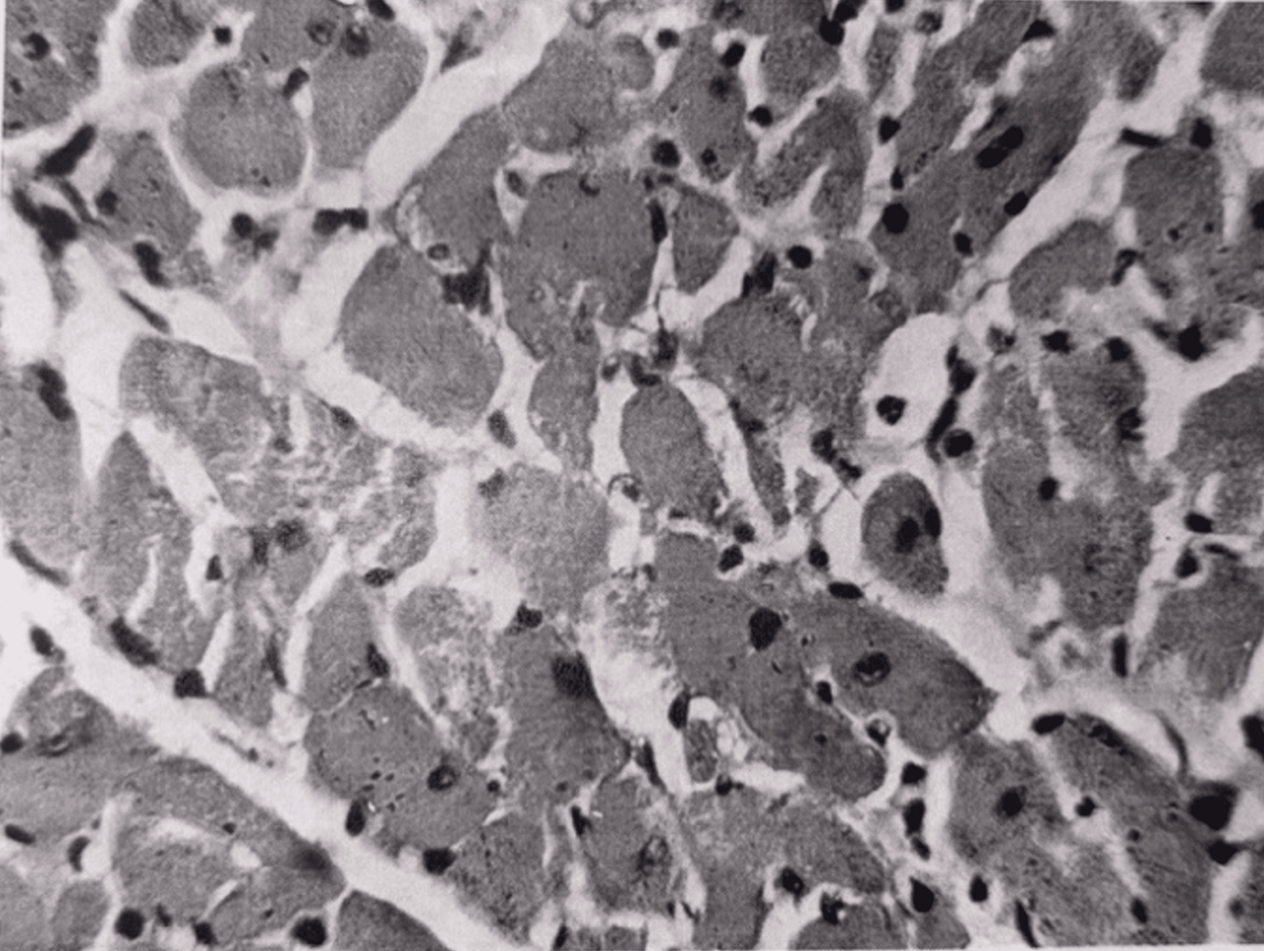
Myocardium 48 hours after restoration of hepatic blood circulation, with underlying cholestasis. Swelling of cardiomyocytes and interstitial edema are observed. Light microscopy, H&E, ×480.

The histological structure of the myocardium was mainly preserved. In some areas, swelling of myocardiocytes and interstitial spaces (Figure 19) and fatty dystrophy were still observed. In some areas, foci of swelling and fragmentation of myocardiocytes were observed, and the striation in the myocardiocytes was erased. The amount of glycogen was reduced compared to normal.

**Figure 20:**
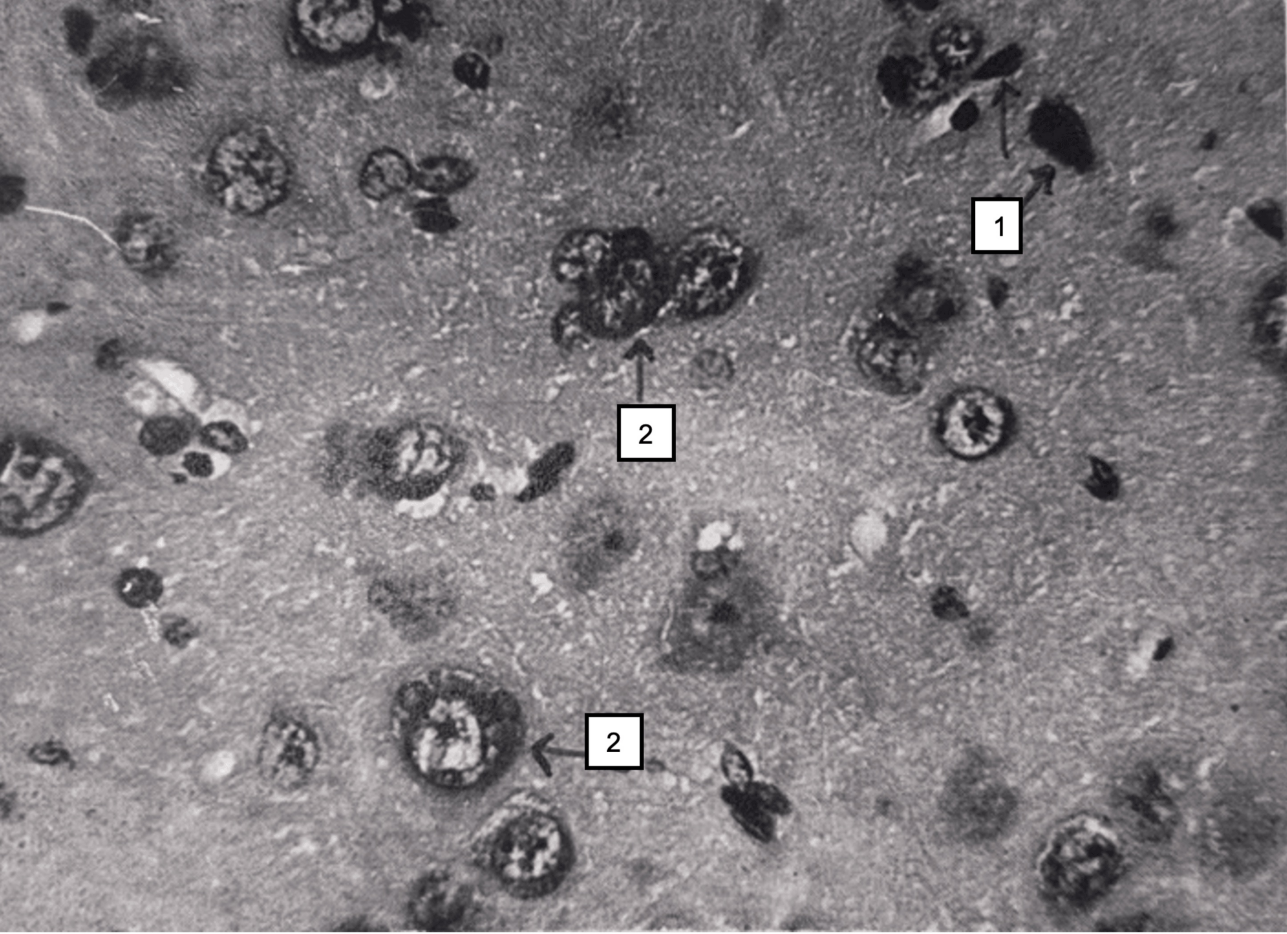
Brain tissue 48 hours after restoration of hepatic blood circulation, with underlying cholestasis. Necrosis of neurons (1) and neurophagia. Light microscopy, H&E, ×480.

Electron microscopy showed destruction of mitochondria in some myocardiocytes, foci of myofibril destruction, deletion of isotropic and anisotropic periodicity, expansion of sarcoplasmic reticulum cisterns, and lipid droplets in some cardiomyocytes. In the brain, pronounced pericellular and perivascular edema was again observed; pyramidal neurocytes of the cortex underwent dystrophy, necrobiosis, and necrotic changes. Fields of neuronal development were observed. The process of neurophagy was visible (Figure 20).

**Figure 21:**
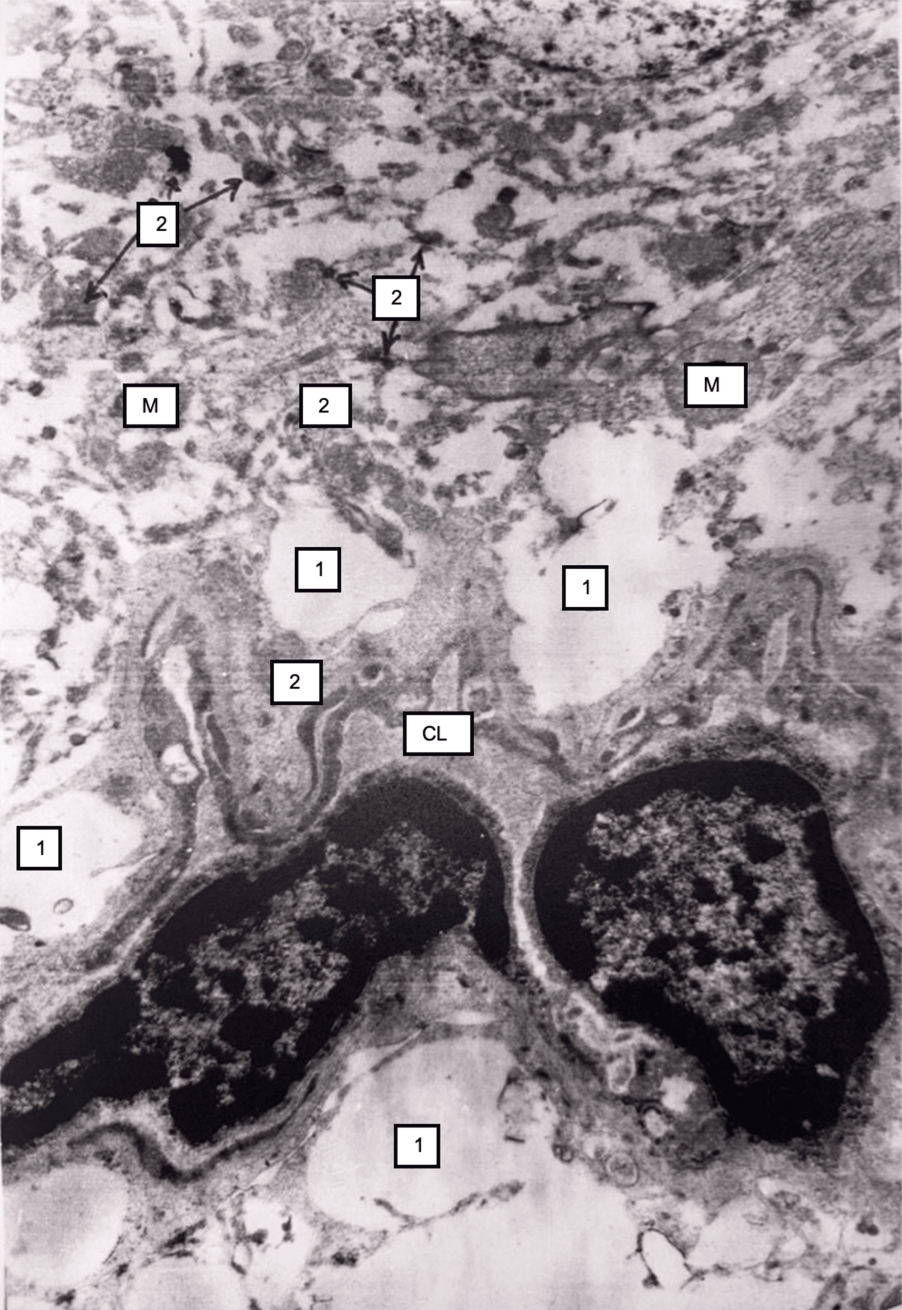
Brain tissue 48 hours after temporary interruption and subsequent restoration of hepatic blood circulation. Pronounced perivascular edema (1) is observed, and the lumen of capillaries (CL) are narrowed. Fragments of desmosomal structures (2) and damaged mitochondria (M) are scattered in the extracellular space. Synaptic structures are disrupted. Electron microscopy, ×15,000.

Electron microscopy showed disorganization of mitochondria, degradation of the rough endoplasmic reticulum, and a decrease in the number of synaptic vesicles in presynapses in the neurocytes of the cortex. Erythrocyte sludge and severe perivascular edema were visible in the lumen of some capillaries (Figure 21).

Results of Morphometric Analysis

The experiment evaluated the area of the bile ducts, their lumens, and the epithelial cells of the bile duct mucosa. Various types of lumens were identified, including circular closed lumens and non-circular open lumens (associated with desquamation of epithelial cells). The ratio between epithelial cells and the bile duct was calculated by subtracting the lumen area from the total bile duct area and dividing the result by the area of epithelial cells. The results of the morphometric analysis are presented in Tables 1-2.

**Table 1:** Morphometric analysis of bile ducts at various intervals following liver ischemia-reperfusion in group without cholestasis (Group 1). Note: The data represent mean values and pertain only to circular bile ducts. Area is expressed in 10^-5 ^mm^2^. Differences are statistically significant (p < 0.05).

Time intervals	Bile duct area	Bile duct lumen area	Epitheliolytic area	Ratio of the cells and bile duct*	P-value
15 min	22.84 ± 8.15	7.56 ± 3.44	2.39 ± 0.46	6.21 ± 0.98	< 0.05
24 h	22.15 ± 6.10	7.60 ± 2.80	2.15 ± 0.30	6.76 ± 0.20	< 0.05
48 h	24.10 ± 5.10	10.30 ± 2.6	5.40 ± 0.25	2.50 ± 0.15	< 0.05

**Table 2:** Morphometric analysis of bile ducts with underlying experimental cholestasis at various time intervals in group with underlying cholestasis (Group 2). Note: The data represent mean values and pertain only to circular bile ducts. Area is expressed in 10^-5^ mm^2^. Differences are statistically significant (p < 0.05).

Time intervals	Bile duct area	Bile duct lumen area	Epitheliolytic area	Ratio of the cells and bile duct*	P-value
15 min	74.22 ± 12.95	8.40 ± 0.60	10.07 ± 2.52	6.53 ± 0.46	< 0.05
24 h	55.55 ± 19.84	9.35 ± 4.20	5.50 ± 1.72	8.40 ± 1.13	< 0.05
48 h	51.30 ± 15.03	11.45 ± 3.15	4.42 ± 1.64	9.00 ± 1.12	< 0.05

In the cholestasis group (Group 2), the total area of bile ducts (74.22 ± 12.95) was nearly 3.5 times higher than in the ischemia-reperfusion group (Group 1) (22.84 ± 8.15). Cholestasis led to massive mechanical dilation of the bile ducts and a strong cellular response, significantly exceeding the changes caused solely by the temporary interruption of blood supply. Statistically significant differences were determined using Student’s t-test (Table 3).

**Table 3:** Results of unpaired two-tailed Student’s t-tests comparing Group 1 and Group 2 at each time point.

Time interval	Bile duct area	Bile duct lumen area	Epitheliolytic area	Ratio of cells to bile duct
15 min	t(12) = 8.88, p < 0.0001	t(12) = 0.64, p = 0.53	t(12) = 7.93, p < 0.0001	t(12) = 0.78, p = 0.45
24 h	t(12) = 4.26, p = 0.001	t(12) = 0.92, p = 0.37	t(12) = 5.08, p < 0.001	t(12) = 3.78, p = 0.003
48 h	t(12) = 4.53, p < 0.001	t(12) = 0.74, p = 0.47	t(12) = 1.56, p = 0.14	t(12) = 15.22, p < 0.0001

Histological specimens clearly demonstrated proliferation of bile duct epithelial cells. This may be caused by increased pressure or activation of certain bile components acting on the cells; however, there are currently no experimental studies confirming this hypothesis.

Our study showed that, based on morphometric data, comparison of two different models of temporary interruption and restoration of hepatic blood circulation with experimental cholestasis revealed that cholestasis induces a more aggressive response, which is particularly evident at the early stage, specifically at 15 minutes.

## Discussion

Characteristic liver changes were observed after knotting and removal of the tourniquet on the hepatoduodenal ligament, without underlying cholestasis, which is manifested by the deepening of damaging changes in the liver at the 24th hour after the restoration of blood circulation, and is explained by the aggravation of ischemic damage to hepatocytes under reperfusion conditions. The rapid rehabilitation of the liver structure and metabolism at the 48th hour is due to the high regenerative potential of this organ. Dyscirculatory and damaging changes in the heart and brain gradually intensify from the 24th hour after the removal of the tourniquet and continue until the 48th hour (when pathological changes in the liver have already been eliminated), which must be due to the so-called "lock-in syndrome," which means that after reperfusion, the microcirculation system of organs damaged by hypoxia is involved in the blood circulation and the transfer of cerebrotoxic and myocardiodepressive factors into the blood [16-20].

In case of underlying cholestasis after temporary disconnection of the liver from the blood circulation and its restoration at different times, the damaging changes in the liver, heart, and brain last longer and are more severe than without cholestasis. Changes in the liver are already visible in the immediate period after the restoration of blood circulation (15 minutes); new formation of bile ducts, or “neoductulogenesis,” is observed [20-26]. In the next 24 and 48 hours, a decrease in the number of neoducts is present, which should be caused by the passage of congested bile into the bile ducts through new paths, drainage of intrahepatic bile ducts, and a decrease in pressure in them [2-4,11,23].

In the brain, as studies have shown, for the entire 48 hours after the tourniquet is removed, there is a pronounced pericellular and perivascular edema and dystrophic changes in neurons with neurophagy. The damaging process is especially severe at 24 hours after the restoration of hepatic blood flow; acute damaging changes are relatively reduced at the 48th hour, but are not eliminated. During this period, areas of neuronal loss are observed. The pathogenesis of the changes that develop in the brain differs from the pathogenesis of the damaging changes in other organs described by us. As is known, the brain is much more protected from ischemia than other organs in conditions of hypovolemia of any genesis. During hypovolemia, when the stroke volume of the heart decreases and the blood pressure decreases, a complex of protective and congestive reactions is triggered in the body, which is manifested by excitation of the sympathetic nervous system and the release of catecholamines and adrenaline, as well as noradrenaline, into the blood in maximum quantities, which is accompanied by spasm of peripheral blood vessels (arterioles). This is accompanied by a redistribution of the circulating blood volume in favor of vital organs; that is, centralization of blood circulation develops. The volume of blood ejected by the heart is distributed unevenly into peripheral fractions. In this regard, the brain is in better condition, although a short-term spasm also develops in the vessels of the brain at the beginning of hypovolemia, which also quickly resolves. The intensification of the damage process in the brain at 24 hours after the restoration of hepatic blood flow, compared to 15 minutes, indicates that the damage to the central nervous system is primarily associated with the intoxication that develops after hypovolemia due to the reperfusion of organs and the release of lysosomal enzymes and other toxic substances from cells and tissues damaged by hypoxia. They contribute to the increase in the permeability of cerebral capillaries and the development of perivascular edema [7].

The heart, like the brain, is a special vital organ; therefore, the changes that develop in it are especially important in the different periods of temporary disconnection of the liver from the blood flow and its restoration [8].

Changes in the heart should be caused mainly by two mechanisms: firstly, a decrease in central venous pressure in the period immediately after the transection of the hepatoduodenal ligament, leading to hypovolemia and impaired coronary blood circulation; and secondly, toxemia that develops in the blood after the restoration of hepatic blood circulation and the appearance of myocardio-depressive factors, both of which are associated with the so-called "engagement syndrome." Therefore, in our findings, both hypoxic and dyscirculatory changes are observed in myocardiocytes. Hypoxic damaging changes in the heart do not reach a critical state due to the centralization of blood circulation, during which the myocardium, as well as the central nervous system, is in a privileged position compared to other organs. Therefore, in the development of primary heart failure, it seems that not only and not so much coronary hypoxia plays a major role, but also cardiotoxic substances that are produced in other organs. Cardiac function can be inhibited by factors such as lysosomal hydrolases and zymogenic granules. Thus, heart failure, which can develop at different times after temporary interruption of hepatic blood flow and its restoration, can be due to both cardiac and extracardiac causes [8]. Based on the presented morphometric data, it can be said that after temporary interruption of the hepatic blood flow and its restoration in two different types and in response to experimental cholestasis, it can be concluded that cholestasis causes a more aggressive reaction, and the most noticeable difference is seen at the initial stage (15 minutes). In the cholestasis group (Group 2), the total area of the bile duct (74.22 ± 12.95) is almost 3.5 times higher than in the ischemia-reperfusion group (22.84 ± 8.15) (Group 1). Cholestasis causes massive mechanical stretching of the ducts and a cellular reaction, which is much stronger than just a temporary interruption of the blood supply [14,15].

Without cholestasis, the area of the duct and epithelial cells increases, which indicates a delayed reactive or regenerative process after the restoration of bile circulation. In cholestasis, there is an initial increase in the total area of the duct and a gradual decrease in the area of the epithelial cells by 48 hours, which may indicate stabilization of acute edema or thinning of the cells under conditions of constant high pressure [6,11].

The ratio of epithelial cells to the duct decreases sharply by 48 hours without cholestasis. Together with the increase in the area of the epithelial cells, this indicates hypertrophy (enlargement) of the cells and structural transformation of the duct wall. In cholestasis, the ratio remains the same and only increases by 48 hours. This means that despite the decrease in the total value of the duct, the epithelial layer maintains a dominant structural role, which is likely a compensatory mechanism to cope with the increased internal pressure [6,11].

The bile duct system is particularly sensitive to pressure changes (cholestasis), which is manifested by immediate dilation (expansion). The reaction to circulatory disorders (ischemia) is slower and is characterized by delayed hypertrophic changes. Both conditions significantly change the histomorphometric picture of the liver, confirmed by a p < 0.05 level of confidence [14,15].

Limitations and future directions

Monitoring Duration

The intervals of 15 minutes, 24 hours, and 48 hours provide an essential look at early reperfusion effects but fail to capture the prolonged recovery or deterioration phases.

Variable Regulation

Factors such as the subjects' age, baseline health, and housing conditions were not strictly regulated, acting as potential confounding influences on the observed outcomes.

Technological Scope

The reliance on traditional histological techniques means that the full breadth of molecular and genomic alterations was not captured. To achieve a more holistic perspective, future research should pair histological data with high-throughput molecular analysis.

## Conclusions

The leading cause of the morphogenesis of the damaging changes in the body and the pathogenesis of the violation of the body's homeostasis after temporary interruption of hepatic blood flow and its restoration is not ischemic damage to the liver, but rather a circulatory disorder of the hypovolemic shock type due to congestion in the portal vein system. This results in hypoxic damage to peripheral organs and the development of the so-called "lock-in syndrome" after reperfusion, with severe disruption of the function of vital organs, the heart, and the brain.

The specificity of organ damage is manifested in the fact that, in conditions of cholestasis, damaging changes in the organs occur earlier after the restoration of hepatic blood flow. Therefore, in the immediate period after the removal of the tourniquet, severe changes occur in the liver, which later undergo regression, although not complete rehabilitation. The primary site of pathological changes shifts from the liver to vital organs such as the central nervous system and the heart.

For the effective clinical application of hepatoduodenal ligament clamping to prevent bleeding during major liver surgeries, we believe that the morpho-functional state of both the liver and extrahepatic organs must be considered preoperatively and intraoperatively. Appropriate prophylactic and therapeutic measures should be implemented, with a high state of readiness to correct functional disruptions in any organ, particularly vital ones. To prevent and treat functional impairment of the brain and heart, detoxification therapy should be utilized.
